# How Advanced Practice Nurses Can Be Better Managed in Hospitals: A Multi-Case Study

**DOI:** 10.3390/healthcare11060780

**Published:** 2023-03-07

**Authors:** Jia Wan, Haiou Xia

**Affiliations:** School of Nursing, Fudan University, Shanghai 200437, China

**Keywords:** labor markets for care professionals, resource orchestration, advanced practice nurse, management strategies, case study

## Abstract

The labor markets for care professionals are a perennial topic of discussion. Advanced practice nurses (APN), as an advanced role in nursing, arose to solve the shortage of primary health care professionals. Prior research has advanced several areas of exploration for APNs’ training or employing methods in Chinese hospitals. However, this leaves a key imperative unexplored: the management strategy of APNs in hospitals. The present study seeks to explore the management strategy of APNs in Chinese hospitals. The resource orchestration theory served as the guide as the multi-case study method investigated 18 case hospitals, gathered information from a variety of case data sources, and summarized the management strategies for hospitals’ advanced practice nurses. Four types of APN management strategies—expert customized type, hierarchical linkage type, multidisciplinary benefit type, and professional penetration type—have been identified through resource orchestration. Hospitals can utilize the APN management strategy model as a guide to manage APNs in accordance with the unique characteristics of APN resources.

## 1. Introduction

Advanced practice nurses (APN) have become a hot topic in recent years, as an advanced role in the labor markets for care professionals [1,2]. An APN is defined by the International Council of Nurses (ICN) as a registered nurse who possesses expert knowledge, sophisticated decision-making skills, and clinical competencies, enabling them to expand their practice certification [3]. Nowadays, over 70 nations have recognized the significance of APNs [4]. Studies have shown that APNs can enhance patient care for those with complex needs [5,6]. What is more, APNs have been demonstrated to have a positive impact on health economics by boosting healthcare usage and reducing costs [5,7].

The majority of nurses in mainland China work in a single hospital throughout their career until they retire [8]. This has led scholars to investigate and focus on implementing APNs in hospitals, providing advanced talent for the labor market for care professionals [9]. However, although it is a common role internationally, the role of an APN is still in its early stages of development in China and lacks a defined description [10]. APNs were first introduced into China in 2000 [2]. As of 2020, the total number of registered nurses in China has surpassed 4.7 million, with over 50,000 having completed APN training, accounting for more than 1% of China’s nursing staff [11,12]. There are now some established and distinct APN patterns. Some hospitals design APN as a higher level than specialist nurse [13,14]. However, there is a lack of consistency in training or evaluation. What is more, there is no unified certification body and standard for APNs, resulting in a disconnect between APNs’ training and employment in hospitals.

Many high-income countries in Western Europe, North America, and Oceania have established and maintained the APN position and its educational requirements through legislation or regulations, ensuring the effective administration and usage of APNs [15,16]. However, there are also challenges in managing APNs in many countries. For instance, many hospitals in Japan have not reached an agreement on how to effectively utilize APNs, leading to inefficient utilization of APNs [17].

APNs’ management and utilization are currently in the exploratory phase in mainland China. There are significant variances in the system needs and norms, and Chinese hospitals develop their own plans for utilizing and managing APNs based on their unique circumstances [10,13]. Several hospitals have limited APNs’ time to focus on specialized work after completing their training, which negatively impacts their professional progress and enthusiasm [18,19,20]. Moreover, research on APN management mostly focuses on the theoretical level and sharing practical experience in specific hospitals or specialty fields, resulting in a lack of unification and high variability [8,21]. Therefore, hospital administrators need to prioritize strategic development when managing APN talent resources. 

Resource orchestration refers to managers’ capacity to actively build, expand, or adjust an organization’s resource base in order to apply strategic resources [22,23]. According to theory, the way resources are managed is just as crucial as the resources themselves. By combining resources, talents, and managerial acumen, the value of resources can be maximized, and a competitive advantage can be maintained [24]. 

The processes of resource orchestration involves three key stages: resource organization, resource bundling, and resource utilization [22]. Resource organization involves the acquisition, accumulation, and stripping of resources. Once resources are obtained, they must be combined or tailored to meet their specific requirements. Resource bundling involves constructing and bundling resources to maximize their value. Finally, resource utilization involves mobilizing, coordinating, and deploying resources efficiently to capitalize on opportunities and maximize their value [22,24]. Resource orchestration theory is based on three major dimensions: breadth (across borders), life cycle (various phases of maturity), and depth (across tiers). Since the focus of this research is on advanced stages of development, the life cycle (time) component will be omitted for the time being. 

According to the resource orchestration theory, hospitals cannot rely solely on having higher-level nurse resources to develop a specialized competitive advantage. It is crucial to accumulate, bind, and effectively utilize human resources. Only through effective management can the full value of resources be realized, ultimately leading to the greatest competitive advantage [22,25].

To summarize, how to manage and utilize APNs remains a significant international concern that needs to be handled. Therefore, this study uses the case study method to identify hospitals’ management strategy models among APNs based on resource orchestration theory at the organizational level, in order to provide guidance for the management of APNs in hospitals.

## 2. Materials and Methods

### 2.1. Study Design 

Given limited empirical evidence, as well as exploratory questions about how APNs are managed, a case study approach was deemed appropriate for the study. Hence, a multi-case study was conducted to investigate the management strategy of APNs in 18 Chinese hospitals. Multi-case studies may comprehend the differences and similarities between instances and examine cross-case analysis data in each case situation and across cases [26]. Moreover, the data generated by multi-case studies are robust and dependable, which can lead to a more persuasive explanation [27]. Furthermore, through “replication logic,” many case studies can strengthen the external validity of case studies and make study results more general and rigorous [26].

### 2.2. Sample Characteristics

The multi-case research adheres to the theoretical sampling and replication logic principles. The selection of cases was based on three screening criteria: 

(1) Informative: The selected cases must have a recorded, typical APN system or standard that serves as a public standard within the hospital. The management method of APNs in the selected case hospitals should differ, providing inspiration and direction for other hospitals’ management modes. 

(2) Representativeness: The selection of case hospitals should be representative and impactful, covering various geographical locations and economic situations. The diversity of hospital locations ensures that they can represent the growth direction of administration and employment of APNs in the region. The clear distinctions in the process of expert nurse management improve the study’s external validity and support the replication logic between instances.

(3) Accessibility: The role of APNs in the hospital should have been established for more than 2 years, and the managers of APNs should have certain experience. The data of the case hospital should be highly available, allowing researchers to investigate the formation mechanism of the management model.

Based on the criteria outlined above, this study identified 18 hospitals in China, with 5 located in the east, 6 in the west, 4 in the south, and 3 in the north. This study uses a typical example to compare the management methods of APNs in these 18 institutions as the unit of analysis.

### 2.3. Data Collection 

This study drew on a wide range of data sources, including the following. (1) In-depth semi-structured interviews: From June 2021 to November 2022, directors of nursing departments or nursing managers in 18 hospitals, as well as APNs in hospitals, were interviewed via online videos or offline interviews to determine how APNs are used and managed in each hospital. With hospital APN management as the topic, the interview should have a thorough grasp of the contents, tactics, formation factors, problems, and other elements of hospital APN management measures. (2) The hospital’s internal archives: this includes the relevant system and management plan of the hospital’s APN management. (3) Extraneous resources: these include research papers, hospital experience sharing, news stories, official website dynamics, social media publicity materials, and so on. (4) Non-participatory observation: this includes observing APNs’ work, participating in APNs’ outpatient services, participating in APNs’ hospital reports, sharing experiences, and other activities to understand the use and management strategies of APNs in the hospital from multiple perspectives. Table 1 outlines the case data sources used in this analysis.

### 2.4. Data Analysis

The data analysis in this research, using a multi-case study design, consists mainly of three major steps: First, an intra-case analysis is performed using case descriptions to examine how hospitals in each case situate APNs and carry out training, utilization, and administration within the restrictions of policies and institutions. Second, in this study, the pattern matching of case studies’ analytic technique is applied. Inter-case analysis is used to examine the similarities and differences in the hierarchical management process of APNs at various hospitals. Finally, an overarching framework is offered, with graphics utilized to aid analysis. 

### 2.5. Reliability and Validity

This study closely adheres to the scientific case study technique and Yin’s approach to assure its validity and credibility [26]. First, the study uses a triangle verification connection, establishes an evidence chain, and enhances construction validity. Second, pattern matching is performed based on the resource orchestration theory and management approach of this study to increase internal validity. Third, the analysis framework of the research uses existing theories, and multi-case study methodology is used to increase external validity. Fourth, before the study begins, the research team creates a thorough research draft, including the theoretical foundation, research design, data collection, and analysis. During the research process, NVivo11 software was used to create a detailed research data database, and the case data were coded sentence by sentence using two-person coding. Inconsistencies in coding were resolved through discussion among the research team. This approach increases the study’s dependability and credibility.

## 3. Results

### 3.1. Research Context

This study has identified significant disparities in APN management practices across hospitals, which can be attributed to differences in resources such as the number of nurses, the ratio of bed-to-nurse, and the number of nurses who have completed APN training. Through an inter-case study of 18 hospitals, the study has identified four types of hospital APN management strategies: expert customized type, hierarchical linkage type, multidisciplinary benefit type, and professional penetration type. Table 2 describes the resource data from 18 hospitals. Appendix A contains detailed data from 18 cases of APN management in Chinese hospitals.

### 3.2. The Four Types of APN Management Strategy

**Figure 1:**
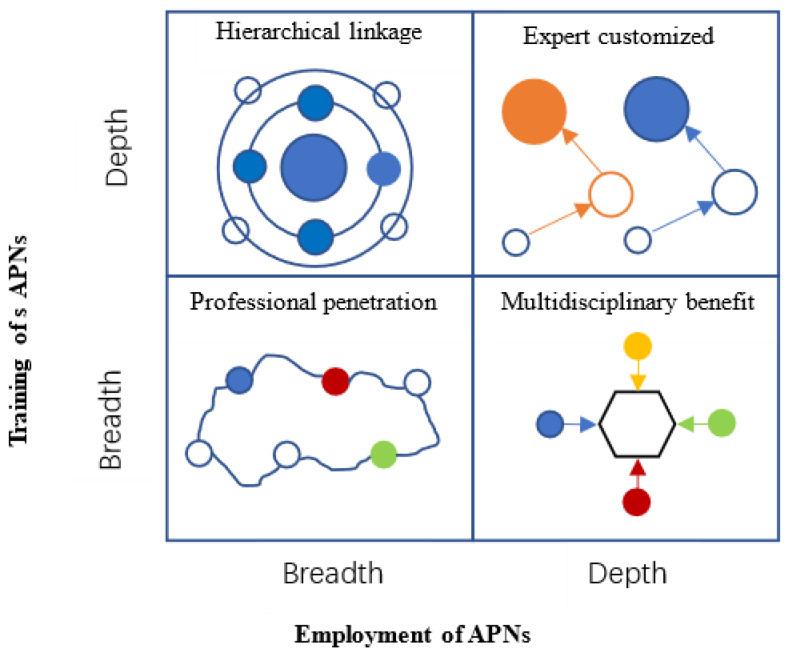
Management strategies of APNs. In the diagram, solid circles indicate APNs, while hollow circles represent general nurses. The size of the circle denotes the amount of training, and the color of the circle symbolizes the various specializations. The hollow hexagon depicts a distinct group of patients who are the attention of APNs.

This study has discovered the management strategies of APNs through case studies of 18 hospitals. The process involves leaders identifying and seizing APN development opportunities through strategic positioning in an uncertain environment. They create the development environment of APNs in hospitals through resource mining and integration, so that APNs can create value and seek growth. This research separates the human resources of APNs into two dimensions based on resource arrangement theory: depth (level and specialization) and breadth (diversity and scope) of people training and employment [22]. Hospital administrators employ the resources of APN training, staff allocation, and management structure to establish four management methods with distinct characteristics: expert customized type, hierarchical linkage type, multidisciplinary benefit type, and professional penetration type (Figure 1). Table 3 provides typical instances of intra-case analysis of the four hospital approach categories. 

#### 3.2.1. Expert Customized Type (Depth Training–Depth Employment)

The expert tailored management plan is intended for hospitals with limited technical strength of specialist nursing employees but enough nurse resource reserves, strong training resources, and high-level talent reserve. When hospital leaders identify clinical needs, they carry out top-level design through resource integration and introduce the APN role that is in line with international standards into the hospital’s practice environment and nursing mode. The expert customized strategy can deeply integrate specialized talent training with talent use, provide highly matched APN experts for the target clinical needs, transform high-level nursing resources into APNs, and become expert leaders in the specialty field. The hospital may continually attract the introduction of high-level nursing talents and create a sufficient reserve for the follow-up hospital APN talents by utilizing the expert customized method. The expert customized method includes the following features:

Depth training includes the following. (1) Customized training: the hospital creates customized pre-service training and follow-up training plans based on distinct post contents in order to give APNs the training required by post competencies. (2) Internship period: The job substance of an APN position differs from that of clinical nurses. As a result, an internship period is necessary to confirm that the APN satisfies the APN competence practice criteria. (3) Discipline diversity and continual self-improvement: This is used to develop APNs in various disciplines. Each post has its own separate post content and duties, and training material and assessment standards are developed to meet the core competency criteria of that post. APNs need to keep learning and developing in accordance with their core competency.

Depth employment includes the following. (1) In-depth post design: Expert criterion, competitive selection, and ongoing career development. Competition for “top tier” posts: nurses who satisfy the recruiting expert standards are eligible to apply, and those who have completed necessary specialty nurse training are favored. Nursing talents whose comprehensive ability may fulfill the competency standards of APNs are picked through the established recruiting method. Following the selection, the hospital will award the APN’s mark to establish their identification (exclusive badge). As experts, the hospital is responsible for attending to and aiding with each APN’s career development goals and professional development requirements. (2) Team management: establish a specialized nursing team lead by an APN to perform expert academic activities, training, and so on. (3) Expert salary standards: APNs should be valued as much as possible. The bonus coefficient standard fits the APN’s post content. (4) Rigorous evaluative criteria: APNs must utilize rigorous evaluation criteria to sustain the continual growth of their role’s core competences and values as an expert in nursing.

Hospital A is a typical instance of expert customized APN management: Hospital A has a bed-to-nurse ratio of 1.08, appropriate nursing human resources, and high-level nursing abilities, and 26.25% of nurses have finished APN training. As a result, the hospital customizes the strategic level of APNs, which is higher than specialized nurses in China, and provides independent positions. Through the customized training for professional nursing skills, APNs will acquire profound professional knowledge, exquisite clinical experience, and complicated problem-solving abilities. APNs practice in 15 specialist practice areas at Hospital A. By expert customized management measures (Table 4), APNs at Hospital A have a strong effect in their specialty area. Furthermore, APN management in Hospital A has become the leading management method in China.

#### 3.2.2. Hierarchical Linkage Type (Depth Training–Breadth Employment)

With the constant expansion in the number of APNs training, hospitals carry out hierarchical management for APNs at the hospital level, considering the demands of professional development of APNs. The hierarchical linking approach enables the formation of talent sequences and a consistent flow of reserve talents, allowing the hospital to spread the risk of brain drain created by the unpredictability of talent development in APNs’ management.

Depth training includes the following. (1) Hierarchical training: The hospital created a hierarchical training approach to foster higher-level specialist nursing abilities. To give advice at the expert level, a higher level of APNs is established above the specialist nurse level. Professional queries can be answered by an APN, allowing APNs to increase their impact in the hospital. (2) Create career path: The professional growth route and the management route are created as two parallel development paths established for the hospital’s nursing staff. For future growth, APNs might pick one of two development paths to establish a clear development direction for career planning. (3) Continual self-improvement and training funding: The hospital provides incentives for APNs, such as training fundings, to encourage them to continue to learn and sharpen their skills.

Breadth employment includes the following. (1) Competitive selection and hierarchical position: nurses who have completed training at different levels need to compete for positions and become APNs at different hierarchical levels. (2) Grid management: Grid management is carried out in the same specialty direction, an APN is designated as the team leader, and expert nurses are disseminated to all hospital departments in need. Departments that do not have specialized nurses might choose a liaison nurse to join the specialty team. The team can lead and manage the expert employees through frequent group activities. (3) Hierarchical salary standards, comprehensive evaluative criteria, and ongoing career development: APNs are managed by hierarchical scales, compensated by different levels, examined and evaluated comprehensively for core skills of positions, and supplied with an ongoing professional growth space in accordance with the career path. 

Hospital B is an example of hierarchical linkage APN management. Hospital B has a bed-to-nurse ratio of 62%, which is significantly low in terms of nursing human resources when compared to Hospital A. The development demands of clinical human resources and APN abilities should be thoroughly examined. A total of 10.65% of the hospital’s nursing staff has undergone APN training. To offer a professional path development ladder for APNs, the hospital established a three-level management mode of nurse–specialist nurse–APN (clinical nurse specialist). Excellent nurses are chosen from the specialty group to become APNs to ensure the long-term development of the specialist group. The hierarchical linkage APN management measures of Hospital B can be seen in Table 5. In the present year, nearly ten APNs have been educated in multiple disciplines, and a special nursing committee has been formed around the APN in the same specialty. The APN directs the work of the committee, and the committee members direct the nursing work in their unit. The establishment of hierarchical links promotes the fast growth of hospital specialties. 

#### 3.2.3. Multidisciplinary Benefit Type (Breadth Training–Depth Employment)

Specialized gain management strategies may be implemented in hospitals with a significant number of APNs in different specialties. The manager can integrate the resources of various specialties and use the diverse specialty platform to form multiple multidisciplinary APN teams. The teams can meet the needs of different patient groups in conjunction with the advantageous disciplines of the hospital. For hospitals, especially at the departmental level, APNs of different specialties focus on specific patient groups, forming a nursing mode of multi-specialty cooperation, which can deeply explore the multi-specialty cooperation effect. APNs operate as collaborators in multidisciplinary teams, offering community health management and long-term illness follow-up while creating specialist care and providing patients with higher quality nursing practices.

Breadth training includes the following. (1) Extensive training and continual self-improvement: The hospital employs a sizable number of nurses and cultivates a diverse pool of skilled APNs. In order to develop the resource integration of the hospital nursing specialty, the hospital accords significant attention to the advancement of the APN subspecialty and encourages its continual self-improvement. (2) APN platform and specialty workshop: to focus on and satisfy the requirements of different patient groups, the hospital employs an open and diversified specialized platform to adapt the APN architecture and build several interdisciplinary APN teams.

Depth employment includes the following. (1) Full-time position, high salary standards, and ongoing career development: Outstanding APNs will be chosen from specialized teams, and full-time positions will be created. Pay is increased and greater professional growth opportunities are provided. (2) Multidisciplinary nursing models and whole life cycle: specialized teams can broaden their work scope to the surrounding region and provide whole life-cycle care, and they establish a high-level interdisciplinary nursing mode for patients. (3) Multidisciplinary APN team: At the department level, APNs from various disciplines focus on specific patient groups, establishing a multidisciplinary APN team. The utilization of multi-specialty collaboration and efficiency is more favorable to providing patients with all-around professional nursing care.

Hospital C is a typical example of the multidisciplinary benefit-type APN management. The hospital boasts a bed-to-nurse ratio of 87% with an abundance of nursing personnel. Additionally, 20.91% of the nursing personnel have undergone specialty nurse training, and the training basis for APNs is extensive, with a significant number of training opportunities available. Table 6 outlines the multidisciplinary benefit APN management measures implemented by Hospital C. The APNs cultivated in Hospital C are distributed across 38 different specialties, allowing for a diverse range of specialized care. The hospital has established a multidisciplinary nursing team for pulmonary rehabilitation and a case management team for lung transplantation, integrating the benefits of respiratory and critical care medicine into their APN management strategy. The pulmonary rehabilitation nursing team consists of 14 nurses from various specialties, who provide patients with comprehensive professional care. The lung transplantation specialist case management team manages the entire lung transplantation process and provides lifelong follow-up care to lung transplantation patients. Moreover, each APN team can extend their scope of work to the community and social level through multi-disciplinary collaboration, forming a distinctive hospital APN atmosphere. Through its effective multidisciplinary benefit-type APN management strategy, Hospital C has achieved a high level of patient care and satisfaction.

#### 3.2.4. Professional Penetration Type (Breadth Training–Breadth Employment)

Nursing human resources are scarce in hospitals that use the professional infiltration management model. More APNs are trained and rapidly enrich the hospital’s APN strength in a short time. This fully exploits the diversity of specialized staff, penetrates APN culture into nurse practice, and strengthens the specialized ability of clinical nursing staff. However, due to a shortage of personnel and other resources, the hospital is unable to provide more posts and support to optimize the potential and worth of APNs. As a result, APNs face challenges in obtaining additional development resources, and the professional development route is rather limited when compared to other management types.

Breadth training includes the following. (1) Extensive training and competitive selection: Nursing human resources are relatively insufficient, so candidates for APN training face competitive election to maximize the utilization of resources. The hospital may quickly enrich the APN team in a short amount of time by relying on the training at all levels and the training of the hospital itself. (2) APN badge and continual self-improvement: APNs are required to wear badges to work. APNs incorporate specialist knowledge and abilities into everyday clinical practice, fully utilize the department’s diverse benefits, and foster the department’s ongoing self-improvement.

Breadth employment includes the following. (1) Part-time position and specialty groups: APNs are not given full-time posts in their specialty, but a clinical nursing professional group is established for part-time APNs. Members of the group share responsibility for resolving complex clinical problems and provide a complementary and cooperative form of treatment with the department’s medical care. (2) Management system, comprehensive evaluative criteria, post-competency evaluation, and particular salary standards: Although there is no full-time post for an APN, the hospital has formed an APN management system to clarify the role of APNs. APN levels are classified according to training level to define distinct job duties and job topics, and their evaluation criteria and salary standards are tied to the levels.

Hospital D is a typical example of professional penetration APN management. The bed-to-nurse ratio in the hospital is 57%, indicating a shortage of nurses there. The professional penetration APN management measures of Hospital D can be seen in Table 7. In terms of APN training, hospitals assign varied job tasks and management tactics to APNs based on multi-level training levels at the provincial, municipal, and hospital levels. The hospital nursing staff has rapidly professionalized and is now capable of seeking breakthroughs in a variety of specialized areas. Every two years, the hospital revises the management system of APNs. At present, the hospital is separated into two-level management. The selection criteria, job duties, wage structure, and evaluation criteria are all unique. Although there are no full-time positions for APNs, their job duties and contents are defined through the management system, and nurse clinics dominated by APNs are set up to broaden their work scope. Eleven clinical nursing professional groups have been established throughout the hospital. The hospital’s wound ostomy team has established the province’s largest wound care facility and gained regional clout in the specialized sector.

## 4. Discussion

The APN is a critical human resource for the advancement of the nursing discipline and profession. Hospital managers need to ensure strategic planning, rational arrangement, and utilization of APNs to maximize the value of resources. The present management strategy of hospital APNs, according to this study, is one of four strategies: expert customized type, hierarchical linkage type, multidisciplinary benefit type, and professional penetration type. The construction of four distinct strategies is due to the fact that the heart of resource arrangement theory is the implementation of managers’ consciousness. Nursing managers must put out the APN’s development strategy, establish an innovative cultivation plan, and coordinate numerous resources throughout specialty planning. The resource orchestration of hospital APNs is completed via the three stages of resource organization, resource bundling, and resource use, and the development of an APN management strategy. Resource structure permits the acquisition and accumulation of resources, whereas resource bundling enables the transformation and integration of resources into valuable and advantageous capabilities. Finally, via sensible employment, the specialty’s talents, resources, and capabilities may be maximized and value produced [25,28].

### 4.1. Resource Organization—Value of Top-Level Design

Resource organization determines the APN management strategy. In the early stages of resource orchestration, top-level promotion and strategic planning of hospital managers, particularly direct supervisors of APNs, plays a critical role in the construction and fast expansion of the hospital specialty environment [22]. According to resource orchestration theory, the influence of managers on resource orchestration has a higher impact on the resource’s ability and value than the resource itself [29]. As a result, an APN management strategy formulated by nursing managers is the key to the development of APNs. Using the hospital with the expert customized strategy as an example, when the number of APNs is gradually increasing and the development of high-level nursing talents is limited, the director of the nursing department introduces the role of an APN in an innovative way, integrates available resources, and constantly promotes the implementation of the role of an APN in the hospital, which leads to the development of nursing professionals. 

It is suggested that managers should coordinate hospital strategic planning in resource organization and form an APN management strategy. They should promote the establishment of hospital APNs and the advancement of the nursing profession. They should direct the department in providing an APN development space and shaping the APN environment.

### 4.2. Resource Bundling—Echelon Construction to Maintain Competitive Advantage

Resource bundling assists informing strategic characteristics and competitive advantages. The resource bundling stage refers to forming a competitive advantage through resource reorganization and transformation [22]. The hospital builds team management and hierarchical tiers in the human resources arrangement for APNs in order to grow and improve the capacity of hospital APNs. The four management types collectively include more than two levels between nurse and APN. APNs’ hierarchical management can not only give a professional growth route for APNs but also allows for the utilization of APN echelons, allowing for more efficient management. A professional growth ladder for APNs can increase APNs’ independence and autonomy, allowing them to better focus on enhancing clinical practice [30]. The three-tier echelon of “nurse-specialist nurse -APN” formed by the case of Hospital B is similar to the level of APNs in Tongji University’s affiliated hospital, which can effectively motivate APNs to promote nursing discipline development [31]. 

It is suggested that the hospital performs hierarchical management on the APNs, and bundles resources to maintain the competitive advantage of resources and continuously enrich the capability and value of resources. 

### 4.3. Resource Utilization—Inadequate Orchestration Limits Development

Resource utilization assists in ensuring the implementation and development of APNs in hospitals. According to resource orchestration theory, managers should constantly modify the resource mix and capacity allocation depending on the internal and external environment and other factors to sustain competitive advantages [28]. Regulations and policies are significant external environments. The ICN (International Council of Nurses) suggests that regulation is critical to APNs [32]. Many high-income countries in Western Europe, North America, and Oceania have established and supported the APN role [15,16]. Hospitals are unable to adequately utilize APN talents due to the influence of current development problems of APNs in China. Key issues include the internal and external environment such as unclear job duties, a lack of autonomy, and a lack of legal protection, which must be addressed right away. This leads to the issue of inadequate resource allocation in hospitals. According to the findings of this study, hospitals with professional penetration strategies have the problem of inconsistent definition, and APN quality is uneven, which is consistent with the findings of Ding et al. [33]. As a result, clinicians and society lack uniform acknowledgment and recognition of APNs, resulting in insufficient allocation of resources in hospitals for APNs. 

It is suggested that the hospital mobilizes, coordinates, and deploys specialized human resources in a reasonable manner through reasonable resource integration, learns from training and uses management methods of other strategy types, and improves the internal environment in order to fully exploit the maximum advantage and value of resources. Furthermore, policies and regulations pertaining to APNs are expected to be implemented as soon as possible in order to improve the external environment for APN applications, ensure the stability of the APN team, break down the barriers of hospital resource arrangement, and gradually bring the development of Chinese APNs in line with international standards.

## 5. Conclusions

This study aims to explore the management strategy of APNs in Chinese hospitals. Using the multi-case research technique and guided by the theory of resource orchestration, 18 hospitals were investigated and assessed. Four strategies for the management of APNs in hospitals were summarized: expert customized type, hierarchical linkage type, multidisciplinary benefit type, and professional penetration type. Hospital leaders use resource organization to carry out top-level design for APNs’ strategic positioning, optimal management strategies based on local conditions, optimize the value of APNs’ resources through hierarchical management, and maximize their advantages and abilities.

This study has limitations due to its cross-sectional research design. The development outcomes of APNs may not be fully reflected in this study, due to restricted access to essential information sources and a lack of engagement of other stakeholders in the system (e.g., physicians, patients). A variety of research approaches can be employed in the future to longitudinally track the development and management outcomes of APNs from the level of hospital organization and associated policies in order to analyze and improve APNs’ training, development, and management in hospitals.

## Figures and Tables

**Table 1:** Data sources.

Strategy Type	Primary Interview Data				Secondary Data	
	In-Depth Semi-Structured Interviews/Times	Recorded Word Count/Thousand	Non-Participatory Observation/Times	Observe Manuscript/ Thousand	Literature and Internal Data	News Reports and Online Resources
Expert customized type	6	65	2	29	3	6
Hierarchical linkage type	11	150	2	25	4	3
Multidisciplinary benefit type	9	109	2	27	5	4
Professional penetration type	27	250	2	16	10	4

The secondary data came from Wechat public platform, Health Circles, Nanfang Daily, SOHU, and the published literature and related information.

**Table 2:** Resource data from 18 cases hospital.

Case	Area	Type	Number of Completed APN Training	Number of APNs	Number of Nurses	Number of Beds	Ratio of Bed-to-Nurse (Number of Nurses/Beds)
1	North	Professional penetration type	2000	NA	3451	5300	0.65
2	North	Professional penetration type	1720	NA	3449	6750	0.51
3	East	Professional penetration type	50	NA	2300	2005	1.15
4	East	Expert customized type	80	17	777	820	0.95
5	East	Hierarchical linkage type	181	10	1700	2750	0.62
6	East	Expert customized type	678	15	2583	2400	1.08
7	West	Professional penetration type	458	1	2563	3500	0.73
8	South	Multidisciplinary benefit type	156	9	2398	2861	0.84
9	South	Multidisciplinary benefit type	300	60	1435	1650	0.87
10	South	Hierarchical linkage type	198	20	1860	2500	0.74
11	West	Expert customized type	1234	30	4700	4300	1.09
12	West	Professional penetration type	244	NA	1500	2200	0.68
13	North	Professional penetration type	400	2	1300	2300	0.57
14	West	Professional penetration type	260	NA	636	1001	0.64
15	South	Multidisciplinary benefit type	290	5	1700	1400	1.21
16	East	Hierarchical linkage type	130	8	3473	4520	0.77
17	West	Professional penetration type	100	NA	1500	3000	0.50
18	West	Multidisciplinary benefit type	300	13	1200	1520	0.79

NA: Full-time APN positions in hospitals have not been established or are unclear.

**Table 3:** Typical examples cited in the case analysis of the four strategy types of hospitals.

Strategy Type	Typical Case	Area	Ratio of Nurses Completed APN Training (Number of Completed APN Training/Number of Nurses)	Ratio of APN (Number of APN/Total Number of Nurses)	Ratio of Bed-to-Nurse (Total Amount of Nurses/Number of Beds)
Expert customized type	A (Case 6)	East	26.25%	0.58%	108%
Hierarchical linkage type	B (Case 5)	East	10.65%	0.59%	62%
Multidisciplinary benefit type	C (Case 9)	South	20.91%	4.18%	87%
Professional penetration type	D (Case 13)	West	30.77%	0.15%	57%

**Table 4:** Typical examples of management strategies of expert customized type.

Strategy		Characteristic	Citing Typical Examples	
**Expert customized type**	**Depth** **Training** **(Level)**	**Customized training**	Participate in advanced nursing practice courses as well as clinical and instructional courses approved by the American Nurses Association (ANA).	
		**Internship** **period**	After 6 to 9 months, new APNs are able to function alone.	
		**Discipline** **diversity**	APNs are trained in different disciplines (stroke, diabetes, mental health, peritoneal dialysis, cardiology, health management, venous and wound ostomy).	
		**Continual self-improvement**	APNs must complete at least 30 h of continuing education training per year (including at least 15 h pertinent to their specialties).	
	**Depth** **Employment** **(Specialization)**	**Expert criterion**	More than 5 years of experience, a master’s degree, and fluency in English.	
		**Competitive** **selection**	Consists of a written test followed by an interview. The written test questions are drawn from the American Nurse Qualification Examination bank in order to assess the English, basic knowledge, and competence level of nursing professionals.	
		**Team** **management**	Every month, the hospital forms a dedicated nursing committee, with an APN at the helm, to provide hierarchical continuous education for clinical nurses in the form of training courses, disciplinary salons, and special seminars.	
		**Expert salary standards**	APN post salaries are created independently based on post content and are not lower than the level of head nurse.	
		**Rigorous** **evaluative** **criteria**	Annually, APNs are assessed using 360° input from self-assessment, peer evaluation, and supervisor evaluation. Work performance, professional ability, teaching level, research performance, self-growth, and academic leadership are among the assessment markers. There is a distribution of assessment scores and performance, promotion, and study abroad connection.	
		**Ongoing career development**	The Nursing Department will address each APN’s career development objectives as well as their individual and professional development requirements with them.	

**Table 5:** Typical examples of management strategies of hierarchical linkage type.

Strategy		Characteristic	Citing Typical Examples
**Hierarchical linkage type**	**Depth** **Training** **(Level)**	**Hierarchical training**	Clinical nurses are trained in layers in the form of hierarchical training, and APNs are trained as clinical nurse specialists.
		**Create career path**	Clinical nurses might pursue a career as a nurse–specialist nurse–APN (clinical nurse specialist).
		**Continual self-improvement**	Consistently promote the education of APNs and encourage more nurses with postgraduate degrees to engage in APN practice.
		**Training funding**	Training funding is given to clinical nurse specialists for continued education and self-improvement.
	**Breadth employment** **(Scope)**	**Competitive selection**	Clear the departmental post demand, and the whole hospital competes for the position of APNs.
		**Hierarchical position**	Each APN position will have a job description created collaboratively by the department director and the head nurse. Job descriptions below the APN were created by the head nurse.
		**Grid management**	Set up specialty committees with APN at its center, including APNs and clinical nurses, and use the committee members to lead the specialty grid management.
		**Hierarchical salary standards**	Clinical nursing experts are treated better than head nurses, whereas specialist nurses are treated better than educational nurses but worse than head nurses.
		**Comprehensive evaluative criteria**	The assessment criteria of “APN competence Requirements” were developed from four perspectives: clinical professional ability, quality management ability, scientific research ability, and teaching ability. Every two years, APNs will undergo a uniform evaluation and review. Only individuals who pass the evaluation will be able to continue working in the following round.
		**Ongoing career development**	The nursing department will review each APN’s career options.

**Table 6:** Typical examples of management strategies of multidisciplinary benefit type.

Strategy		Characteristic	Citing Typical Examples
**Multidisciplinary benefit type**	**Breadth** **Training** **(Diversity)**	**Extensive training**	Hospital administrators grant an annual nursing development budget to encourage the training and development of APN skills.
		**Continual self-improvement**	The hospital offers several possibilities to participate in high-level APN training, Hospitals give possibilities for APNs to further their education.
		**APN platform**	The nursing director puts everyone in their place and provides APNs with a platform.
		**Specialty workshop**	Organized to exchange new technologies, impart new ideas, and share cutting-edge knowledge of fields.
	**Depth** **Employment** **(Specialization)**	**Full-time position**	Fill about one-fifth of the full-time roles for APNs.
		**Multidisciplinary nursing models**	APNs lead teams to the heart of the community to deliver respiratory and stoma assistance. Through nursing consultation, professional ward circle discussion, specialized outpatient, and other interdisciplinary models, APNs play a key role in the multidisciplinary collaborative nursing mode.
		**Multidisciplinary APN team**	The pulmonary rehabilitation specialist nursing team is made up of multiple APNs who are involved in various elements such as respiratory, intensive care, geriatrics, nutrition, and so on. Patients will benefit more from the presence of multidisciplinary APNs.
		**Whole life cycle**	The case management team follows lung transplant patients throughout their entire life cycle, from the perioperative period to the intensive care period to the rehabilitation period, including full professional nursing visits, outpatient visits after discharge, and lifelong follow-up of lung transplantation patients via Wechat, video, and other means.
		**High salary standards**	Special post APNs are treated in the same way as head nurses.
		**Ongoing career development**	Managers guide APNs in their ongoing professional development. The nursing department’s director offers a platform for APNs to advocate for their positions and rights, as well as to maximize their benefits.

**Table 7:** Typical examples of management strategies of professional penetration type.

Strategy		Characteristic	Citing Typical Examples
**Professional penetration type**	**Breadth** **Training** **(Diversity)**	**Extensive training**	APN training in different levels: ”APN above provincial level” and “Hospital APN level”.
		**Competitive selection**	Participation in national APN training necessitates a unified screening by the nursing department. They must fulfill the selection requirements for APNs and be approved by the department in order to participate in the training of further levels of APNs.
		**APN badge**	APNs who have completed training above the provincial level should wear the APN badge.
		**Continual self-improvement**	Encourage APNs to attend meetings, exchange information, and engage in ongoing education and training.
	**Breadth employment** **(Scope)**	**Part-time position**	APNs work part-time after completing their training, mostly executing their own work and that augmented by APNs’ work.
		**Specialty groups**	The group consists of APNs and department liaison officers, with one member from each unit. Group members conduct hospital-wide consultations and specialist consultations.
		**Management system**	They must perform the post tasks of APNs as specified in the APN management system, and the department will allot specific time to conduct the job of APNs.
		**Comprehensive evaluative criteria**	A three-year tracking, evaluation, and assessment, as well as an annual year-end report and technical and theoretical assessment.
		**Post-competency evaluation**	APNs will be subjected to a post-competency review; a qualification review will be conducted to examine the ability of APNs. If they fail the evaluation, their APN treatment would be canceled. Following the training, they will retake the evaluation the following year, and the relevant APN treatment will be reinstated.
		**Particular salary standards**	A particular level coefficient should be applied to the performance distribution of APNs above the provincial level.

## Data Availability

Not applicable.

## Appendix A

**Table A1:** Data of the 18 cases of APNs Trained and Employed in Chinese Hospitals.

Case	Area	Type	Number of Completed APN Training	Number of APNs	Number of Nurses	Number of Beds	Bed-to-Nurse Ratio (Total Number of Nurses/Number of Beds)	Case Data
1	North	Professional penetration type	2000	NA	3451	5300	0.65	Grade three A general hospital. As a training base for emergency nurses, operating rooms and rehabilitation nurses of China Nursing Association (outside Beijing), nursing and technical training base of China Cardiovascular Health Alliance, and national standardized training base for cardiopulmonary rehabilitation nurses, it undertakes the training and teaching of APNs and a large number of national and provincial training courses for APNs every year.
2	North	Professional penetration type	1720	NA	3449	6750	0.51	Grade three A general hospital. College degree or above accounts for 98.9% of the nursing staff. There are 19 kinds of APNs in the hospital. In 2020, the hospital was approved as the clinical teaching base for APNs in nursing management, neonatal nursing, pediatric nursing, and rehabilitation nursing of the Chinese Nursing Association. As early as 2005, the hospital fully implemented the vertical management of nursing, through the construction of responsibility, rights, interests of the unified vertical management system of nursing, to maximize the enthusiasm of nursing staff, and explore a scientific and effective nursing management mode.
3	East	Professional penetration type	50	NA	2300	2005	1.15	Grade three A general hospital. A total of 70% of clinical nurses had a bachelor’s degree or above, 3% had a master’s degree, and 5 had a doctor’s degree. It is the clinical teaching construction base for blood purification, emergency, and cardiovascular nurses of the Chinese Nursing Association in Beijing. The hospital is the training base for intensive care, hemodialysis, emergency, operating room, ostomy, and community diabetes nurses of the provincial nursing society. The Nursing department undertakes the training and further study of hundreds of specialized nursing talents from all over the country every year.
4	East	Expert customized type	80	17	777	820	0.95	One of the oldest maternity hospitals in China. They pay attention to the training of APN talents, and set up 6 APN teams in the form of academic groups, including a midwifery group, breastfeeding group, tumor nursing group, psychological nursing group, nutrition-related group, and neonatal nursing group. The number of sub-specialist team members based on APNs has steadily increased and become more mature, and the post of APNs has been set up in a pioneering way. In the process of team building, several sub-specialty nursing research directions were gradually condensed to form the characteristics of sub-specialty nursing in obstetrics and gynecology.
5	East	Hierarchical linkage type	181	10	1700	2750	0.62	Grade three A general hospital. The Nursing Department has established academic groups such as “Critical Illness Management Committee”, “Intravenous Infusion Management Committee”, “pressure ulcer Management Committee”, and “Pain Nursing Committee”. Through the operation form of the special committee, the academic level is improved and the development of the nursing specialty is promoted. At present, they have trained more than 10 APNs in the fields of diabetes, ostomy-wound incontinence, PICC, blood purification, critical illness, etc. It is the training base of emergency first aid, critical care, operating room, ostomy school, and other specialties of the provincial nursing society.
6	East	Expert customized type	678	15	2583	2400	1.08	Grade three A general hospital. The nursing department was controlled vertically when the hospital was constructed. Nursing staff educational structure: doctorate and master’s degree 4.5%, bachelor’s degree or above 97.6%. Since 2000, Advanced Nursing Practice has been gradually carried out, and selected key clinical nurses are uniformly named Advanced Practice Nurses (APN). As one of the first hospitals in mainland China to set up APNs, the hospital has experienced the admission, management, practice, and evaluation of APNs in the past 20 years. The hospital undertakes national and provincial training and practical teaching bases for intensive care nurses, such as the Clinical teaching base outside Beijing of the Chinese Nursing Association. At present, there are 15 APN positions in the hospital (stroke nurse, education nurse, diabetes, mental health, peritoneal dialysis, cardiology, health management nurse, venous and wound ostomy).
7	West	Professional penetration type	458	1	2563	3500	0.73	Grade three A general hospital. The hospital has 100 nursing units, 5 nurse doctors, 9% master’s degree, and 72.95% bachelor’s degree or above. NP: 1 person. The hospital undertakes 23 provincial training and practice bases for APNs and 10 national training bases. The International training base of ostomy Therapist School, national clinical practice training base for dental nurses, etc., have formed specialties/special diseases or special nursing with distinct characteristics such as emergency care, intensive care, intravenous therapy, wound stomy, diabetes, VTE, nutrition support, clinical alarm management, etc. It took the lead in setting up the joint clinic of nutrition consultation and medical care for diabetic foot, vascular ulcer of lower limb, and kidney disease in China. At present, it has set up 17 specialized nursing clinics, forming a nurse-led continuous service from inpatient to outpatient, hospital to family.
8	South	Multidisciplinary benefit type	156	9	2398	2861	0.84	Grade three A general hospital. There are 99 nursing units, 98.25% of nursing staff have college degree or above, 11 have a master’s degree. It has 9 full-time specialized APN posts (2 were set up in 2009, for diabetes and wound ostomy; in 2016, 7 additional posts were added, including 3 in ICU units, 1 in the geriatric unit, 1 in the orthopedics unit, and 2 in operating rooms).
9	South	Multidisciplinary benefit type	300	60	1435	1650	0.87	Grade three A general hospital. With a bachelor’s degree or above accounting for 82%, 13 specialist groups have been set up to lead the professional development. In recent years, according to the development requirements of high-level hospitals, backbone nurses are regularly selected and sent to Britain, Singapore, Hong Kong, Macao, Taiwan, Beijing, Shanghai, and other places to gain advanced nursing experience. Providing the platform for clinical evidence-based nursing practice, teaching, and scientific research for APNs. Gives full play to its clinical guiding role, improves the quality of specialized nursing and the level of the whole nursing discipline, and reflects the professional value of nurses. The hospital has obtained 47 training bases for APNs in national, provincial, and municipal departments such as pulmonary rehabilitation nursing, cardiopulmonary rehabilitation nursing, critical care nursing, emergency nursing, surgical nursing, venous therapy, wound ostomy treatment, clinical nursing education, etc.
10	South	Hierarchical linkage type	198	20	1860	2500	0.74	Grade three A general hospital. One person has a doctor’s degree, 1.8 percent have a master’s degree, and 55 percent have a bachelor’s degree or above. The hospital was one of the first national clinical key specialty construction projects in the nursing specialty. In 2020, intensive nursing, emergency nursing, and diabetes nursing were awarded as clinical teaching bases outside Beijing by the Chinese Nursing Association. The hospital attaches great importance to the construction of specialized nursing. There are 22 nursing specialties and 11 nursing clinics, with an annual service volume of more than 17,000 people. Every year, more than 140 out-of-hospital extended nursing services are provided, serving more than 10,000 people.
11	West	Expert customized type	1234	30	4700	4300	1.09	Grade three A general hospital. An APN training base was established in 2010, and was approved as one of the first batches of national clinical key specialties in 2011. In 2021, a curriculum system based on the six core competencies of APNs was jointly developed with experts from Taiwan, China, to carry out a series of APN training and APN cultivation in six aspects: nursing ability, teaching ability, consulting ability, coordination ability, leadership ability, and research and development ability.
12	West	Professional penetration type	244	NA	1500	2200	0.68	Grade three A general hospital. In 2010, it was approved as one of the first provincial training bases for APNs (intensive care, operating room, and emergency). It was the first to start nursing specialized clinics in municipal hospitals (PICC maintenance, diabetes consultation, wound, ostomy, cardiac rehabilitation, breastfeeding, etc.). In 2019, it was the first to set up provincial ostomy professional studios and postpartum rehabilitation professional studios.
13	North	Professional penetration type	400	2	1300	2300	0.57	The largest third-class A hospital in Northwest China, with 70 hospital nursing units. ICU nurse training started in 2007. The hospital undertakes provincial nurse training and is a practice base, as well as the national training base of the international ostomy Therapist School. The hospital places a high value on the development of nursing talent and the advancement of the nursing profession. ICU APN training began during the “11th Five-Year Plan” era. Provincial APN training and practice bases for geriatric and rehabilitation, as well as national training bases for the international ostomy therapist school, anesthetic, ostomy wound, diabetes, hemodialysis, cardiovascular, and other national training bases, are all provided by the hospital. It has established nurse clinics controlled by APNs, such as an ostomy clinic, an incontinence clinic, PICC maintenance, and diabetes education, as well as 11 clinical nursing professional groups throughout the hospital. The regional specialist nursing practice has adopted the standardized professional management and operation mechanism.
14	West	Professional penetration type	260	NA	636	1001	0.64	Third-class A TCM hospital. Nursing discipline is the national clinical key specialty, the State Administration of Traditional Chinese Medicine “Twelfth Five-Year” is a key specialty, and it is the city’s traditional Chinese medicine hospital in the national nursing key specialty project. The hospital has 30 nursing units, 2% postgraduates, and junior college nurses account for more than 96% of the total nurse team. A mid- and long-term nursing talent development plan has been formulated, and eight nursing professional groups, including critical care nursing, have been established to build a career development platform for training APNs.
15	South	Multidisciplinary benefit type	290	5	1700	1400	1.21	It is the largest third-class A women’s and children’s hospital in South China. It implements the vertical nursing management system. A total of 2.63% of nursing staff had a master’s degree or above, and 80.78% had a bachelor’s degree. The hospital has carried out an overall layout plan in the development of APNs. Taking the operating room specialty as an example, it has expanded this to the construction of several sub-specialties, such as pain, sedation, intensive care unit of anesthesia, etc., and set up a specialist team in the hospital.
16	East	Hierarchical linkage type	130	8	3473	4520	0.77	Grade three A general hospital. Clinical nursing has become the first batch of key clinical specialties of the Ministry of Health. Bachelor’s degree or above 88.2%, master’s degree or above 2%. The hospital has 8 APN posts and assumes the responsibilities of the head of the hospital’s APN team. These include a skin group, wound ostomy, venous therapy, diabetes, weight loss, newborn, breast, gastric dynamics, and other specialty areas.
17	West	Professional penetration type	100	NA	1500	3000	0.50	Third-class A TCM hospital. Nursing staff with a graduate degree or above accounted for 2%. In 2014, the hospital nursing team successfully applied for the national Training Base for TCM nursing backbone talents, which is the only training base in southwest China. In 2019, it was approved as the first provincial APN training base for TCM. In 2020, it was approved as the Chinese Nursing Association’s Practice Base for TCM Treatment nurses outside Beijing. The TCM nursing clinic was officially opened in June 2021.
18	West	Multidisciplinary benefit type	300	13	1200	1520	0.79	It is the largest tertiary A tumor hospital in southwest China. The hospital attaches great importance to the cultivation and construction of APNs. Since 2010, APNs have been cultivated and positions of APNs have been set up, including venous catheterization, wound ostomy, rehabilitation, psychology, nutrition, chronic disease management, etc., and specialist studios for management have been set up, and six studios have been approved by the provincial Health Commission of the first batch of nursing studios.
